# Recent Advances in The Polymer Dispersed Liquid Crystal Composite and Its Applications

**DOI:** 10.3390/molecules25235510

**Published:** 2020-11-25

**Authors:** Mohsin Hassan Saeed, Shuaifeng Zhang, Yaping Cao, Le Zhou, Junmei Hu, Imran Muhammad, Jiumei Xiao, Lanying Zhang, Huai Yang

**Affiliations:** 1 Key Laboratory of Polymer Chemistry and Physics of Ministry of Education, Department of Materials Science and Engineering, College of Engineering, Peking University, Beijing 100871, China; mohsin@pku.edu.cn (M.H.S.); 1701111605@pku.edu.cn (Y.C.); zhoule@pku.edu.cn (L.Z.); imrankhanization@pku.edu.cn (I.M.); 2 Beijing Advanced Innovation Center for Materials Genome Engineering, Department of Materials Physics and Chemistry, School of Materials Science and Engineering, University of Science and Technology Beijing, Beijing 100083, China; shuaifengustb@163.com (S.Z.); junmeihu2019@163.com (J.H.); 3 Department of Applied Mechanics, University of Sciences and Technology Beijing, Beijing 100083, China; jiujiu@sas.ustb.edu.cn

**Keywords:** polymer-dispersed liquid crystals, polymerization induced phase separation, doping, applications

## Abstract

Polymer dispersed liquid crystals (PDLCs) have kindled a spark of interest because of their unique characteristic of electrically controlled switching. However, some issues including high operating voltage, low contrast ratio and poor mechanical properties are hindering their practical applications. To overcome these drawbacks, some measures were taken such as molecular structure optimization of the monomers and liquid crystals, modification of PDLC and doping of nanoparticles and dyes. This review aims at detailing the recent advances in the process, preparations and applications of PDLCs over the past six years.

## 1. Introduction

**Figure 1:**
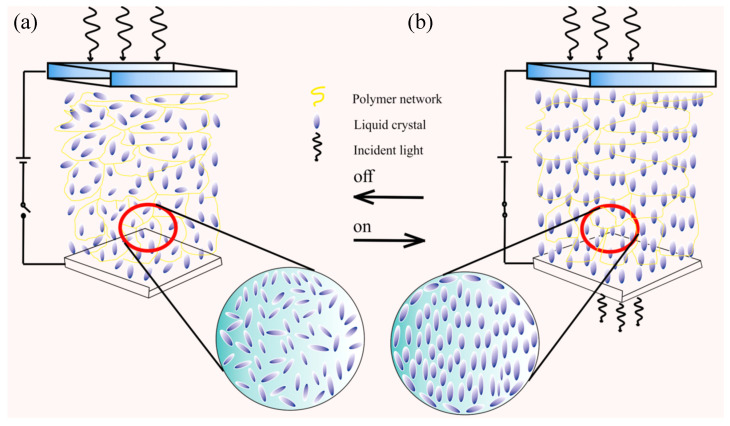
Operating principle of a common polymer dispersed liquid crystal (PDLC) device. (**a**) off-state, (**b**) on-state.

Polymer dispersed liquid crystals (PDLC) were identified as electrically switchable materials four decades ago and have since then remained a topic of intense scientific curiosity. PDLC composite films consist of micrometer or nanometer size liquid crystal (LC) droplets embedded in a polymer matrix. In general, these films exhibit a milky white scattering state due to the random orientation of LC droplets in the polymer matrix [1,2,3]. Once an electric field of sufficient strength is applied, the film becomes transparent and this phenomenon is attributed to the alignment of LC droplets along the direction of the electric field (Figure 1), if the ordinary refractive index n_o_ of LC matches with the refractive index n_p_ of the polymer matrix [4]. Spurred by the employment of PDLC in display technologies, research on these materials has rapidly grown in recent decades and is now extending into areas beyond smart windows [5] and displays [6], including diffuse film [7], antipeeping film, quantum dots (QDs) film [8] and components of organic light emitting diode (OLEDs) [9], field effect transistors (FETs) [10], energy storage [11] and solar-energy harvesting [12].

PDLCs can be prepared by emulsion or phase separation methods [13,14,15]. However, the latter is widely reported in the literature as it allows great control of the morphology, and thus the characteristics, of the final film. This method can be induced by thermal action, solvent evaporation and/or monomer polymerization. The formed LC droplets exhibit great variation depending on the method of phase separation employed. For thermally induced phase separation (TIPS), LC is mixed with a polymer at high temperature, then the mixture is allowed to cool at a specific rate to cause phase separation and, as the polymer hardens, the LC domains appear. For the solvent-induced phase separation (SIPS) process, both the LC and polymer are dissolved in the same solvent. The evaporation of solvent at a specific rate induces phase separation. For the polymerization-induced phase separation (PIPS) method, the LC is mixed with a monomer/prepolymer solution to form a homogenous solution. As the polymerization continues the polymer and LC separate from each other by liquid-liquid or liquid–gel phase [16,17]. The LC molecules come out of the solution and form droplets that grow until polymerization is finished, i.e., when the polymer matrix becomes solid enough [18]. The PIPS method could be further divided into thermal-initiated polymerization and ultraviolet (UV)-initiated polymerization. Although the former method offers good processability, less contamination and strong bonding energy, it requires high temperatures and long duration [19,20]. The latter, however, is prompt, solvent-free and ecofriendly for the production of PDLC films [21].

Despite the stability and durability of the PDLC films prepared by UV- polymerization, nuances in UV-irradiation, molecular weight and solubility of the constituting LCs and polymers could lead to distinct morphologies [22]. Thereby, degree of phase separation and rate of polymerization are influences which control the morphology of LC droplets. Inside the LC droplet, the director configuration is mainly determined by boundary conditions (surface anchoring), LC material parameters and droplet shape and size. External factors (electric or magnetic fields, temperature, stress and so on) change the orientational structure which affects the light-scattering by LC droplets and, consequently, the light transmission of the PDLC film [23,24,25,26,27,28,29,30]. In addition, a slight difference in the refractive index (RI) of both the phases (LCs and polymer) has a significant effect on the light scattering properties of the PDLC films. Moreover, the film thickness also crucially impacts the morphology and electro-optical (E-O) properties of the PDLC films [31]. Studies on these issues can be useful in developing PDLC films for typical applications.

**Figure 2:**
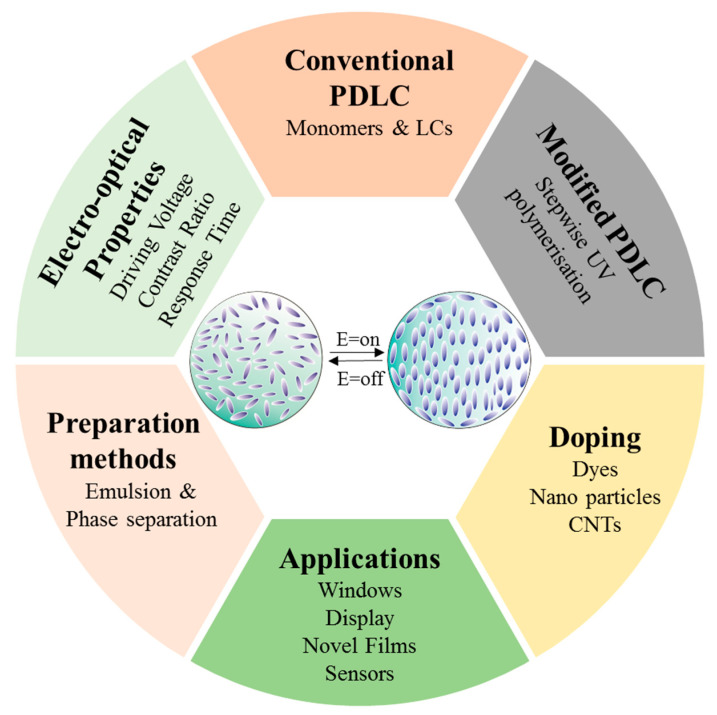
An illustration of the preparation, electro-optical properties optimization and applications of PDLC.

Here, we review the recent developments in preparation, experimental investigation and application of PDLCs, and focus specifically on UV-polymerization. Instead of going into deep details, only specific recent papers between 2014–2020 which are particularly related to UV polymerization, modification techniques related to the process and material to enhance the E-O properties of PDLCs (Figure 2) are cited. However, some papers were exceptionally important and are cited. Furthermore, previous reviews related to these materials [32,33,34,35,36,37,38] can be referred to gain a full understanding of the topic.

## 2. Preparation of Conventional PDLC Composite Film

(Meth)acrylate monomers are generally utilized to prepare PDLC films by UV-polymerization [39]. The modulation of the morphology and E-O properties are limited in the flexible (meth)acrylate monomers. Therefore, some unique structures such as hydroxy, epoxy, branched methylene/methyl, cyclic methylene, phenyl and bisphenol were incorporated into the conventional acrylates [40,41,42,43,44]. Once these structures were incorporated into the polymer matrix, the E-O properties of PDLC composite films could be optimized by varying the hydrogen-bond interaction, the viscosity of acrylate monomers, the refractive index of polymer matrix and polymer network morphologies [45]. Among them, the presence of the hydroxyl group promoted the formation of a thin polymer layer at the surface of PDLC films even at low LC loadings, attributed to improving the E-O properties [46]. Recently, a low-voltage driven PDLC system was achieved through an elegantly simple and uniquely designed acrylate monomer (A3DA) featuring a benzene moiety with a dodecyl terminal chain [47]. In our recent study, we focused on the comparison of methyl and acrylate monomers as well as terminal structures [48]. We found that the asymmetrical substitution of the methyl group on the quaternary carbon in the main chain increased the steric hindrance which reduced the polymerization rate. The contrast ratio (CR) was improved many-fold by changing the monomer structure from flexible to rigid. Similarly, when the monomer with siloxane was incorporated into the polymer matrix, the mechanical properties were enhanced without sacrificing the E-O properties [49]. Table 1 lists some of the (meth)acrylate monomers employed for the preparation of PDLC composites.

PDLC films composed of thiol-ene monomers are usually prepared by UV-polymerization. Norland optical adhesives are good examples of these kinds of monomers. These monomers have high conversion efficiency, fast speed, water resistance, excellent thermal insulation, better inertness to oxidation and less light initiator dosage than the acrylates system [52,53,54]. The thiol-ene reaction usually occurs in two ways: the radical-mediated anti-Markovnikov addition and the base or nucleophile-initiated thiol-Michael addition. In the former, the initiator absorbs energy from light or heat and produces free radicals to initiate the reaction [53]. However, the latter employs a base or a nucleophile which could weaken the bond energy of the alkene double bond to start the reaction in mild conditions. In China, Wang et al. [55,56,57,58] from Sichuan university, in their various studies, employed the thiol-Michael addition to produce dye-doped PDLC films with low driving voltage (V_dr_). In our group Zhong et al. [16] employed vinyl ether and thiol to fabricate PDLCs via the Markovnikov reaction and investigated the morphology and electro-optical properties of the films. Zhang et al. [59] demonstrated that the thiol–acrylate systems could be used to produce PDLC films with higher CR, but the driving voltage was also high. Sun et al. [60] studied the effects of various acrylate monomers with thiols and optimized the microstructure which improved the CR and driving voltage of the PDLC films.

Unlike the previous works focusing on the structures of the monomers, Zhang et al. [61,62,63] from University of Science and Technology, Beijing, China, systematically investigated the effects of LC molecule structures (fluorinated LC molecules, alkene-terminated LC molecules, and cyano-terminated tolane LC molecules) on the morphology and E-O properties of PDLC composite films. The LC component was obtained by doping fluorinated LC molecules into commercial LC (E8) and fixed 50.0 wt% in PDLC composite films. The doping contents and the terminal chain lengths of the fluorinated LC molecules influenced the physical properties (such as dielectric anisotropy, refractive index and viscosity) of LC and polymer network morphology and the E-O properties of PDLC composite films. The results showed that the optimal 8.0 wt% doping fluorine LC molecules enabled them to realize the significantly low driving voltage of the composite films. According to the above study methods, the optimal E-O properties of PDLC composite films with low driving voltage, high CR and fast response time were obtained by employing alkene-terminated LC molecules or cyano-terminated tolane LC molecules.

## 3. Preparation of Modified PDLC Composite Film

**Figure 3:**
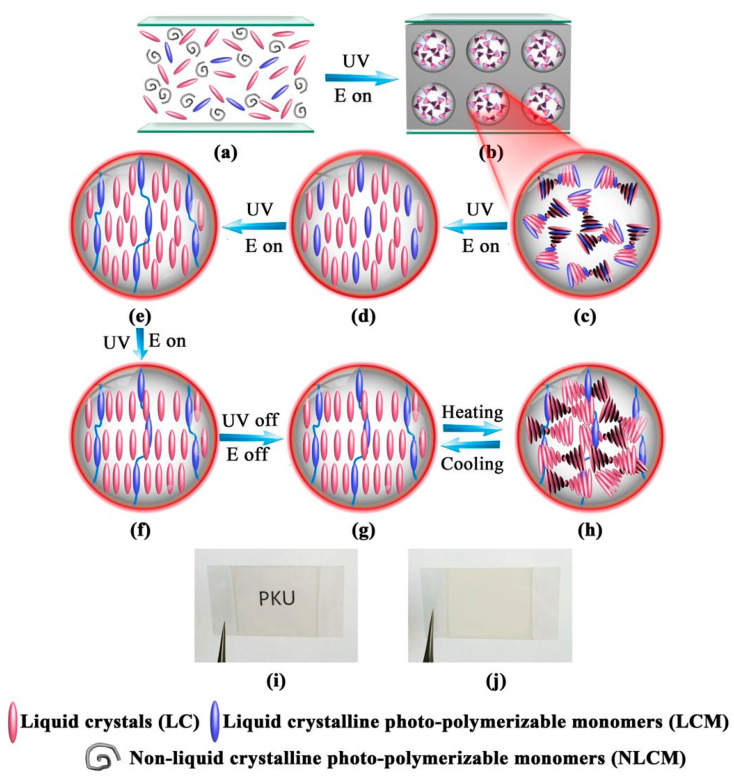
Schematic illustration of the preparation of polymer dispersed and polymer stabilized liquid crystal (PD&PSLC) composite film. (**a**) Homogeneous isotropic mixture sandwiched between two pieces of plastic sheets. (**b**) Microphase separation between the liquid crystalline mixture and polymer matrix. (**c**) Randomly oriented liquid crystalline mixture within a liquid crystal (LC) droplet. (**d**) Perpendicularly aligned liquid crystalline mixture. (**e**) Homeotropically aligned polymer network (HAPN) formed within an LC droplet. (**f**) The N* phase gradually turns into the SmA phase upon the consumption of photopolymerizable monomers. (**g**) Perpendicularly aligned SmA phase within an LC droplet. (**h**) Focal-conic texture of the heat-induced N* phase within an LC droplet. (**i**) Photograph of the transparent state of the film at a temperature below the phase-transition temperature of the LC. (**j**) Photograph of the heat-induced light-scattering state of the film. Reproduced with permission from ref. [66]. Copyright 2017, ACS Publication.

**Figure 4:**
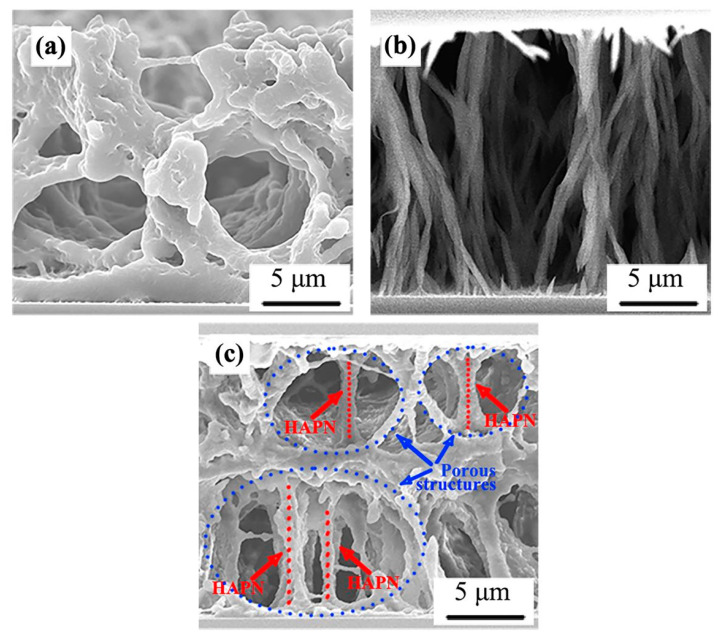
Scanning electron microscope (SEM) photographs of the polymer networks of the films observed from a side view of the cells. (**a**) A porous structure of polymer networks of PDLC composite film. (**b**) The HAPN of PSLC composite film. (**c**) A coexistent structure of both the porous polymer networks and the HAPN of PD&PSLC composite film. Reproduced with permission from ref. [66]. Copyright 2017, ACS Publications.

In the past few years, the modified PIPS technique suggested by Chidichimo et al. [64] of Calabria University, Italy, was further developed as a dual-step polymerization by our research group [65,66,67,68,69,70]. PDLC composite films could be prepared by UV-UV, UV-thermal, thermal-UV or thermal-thermal polymerization in two steps. Liquid crystalline acrylate, vinyl-ether, or epoxy monomers were frequently used. Among them, radical-initiated liquid crystalline acrylate and cationic-initiated liquid crystalline vinyl-ether were usually used for UV polymerization, while liquid crystalline epoxy initiated by UV or thermal could be used for UV or thermal polymerization. Here, a UV-UV dual step polymerization method was taken as an example to introduce the specific method [66]. Figure 3 shows a schematic illustration of the preparation of polymer dispersed and polymer stabilized liquid crystal (PD&PSLC) composite film. The key factor was the lower polymerization rate of the liquid crystalline monomer (LCM) with a rigid structure than that of non-LCM. Thus, the non-LCM radical polymerization was dominant. Further, the microphase separation between LC and the polymer matrix occurred and a porous polymer network structure was formed in the first step, which was similar to PDLC. Subsequently, under sufficient applied electric field and appropriate UV intensity conditions, the oriented LCM was further polymerized to construct the homeotropically aligned polymer network (HAPN) in larger porous structures as shown in Figure 4c, similar to PSLC. The resulting composite film was referred to as PD&PSLC. In the said work, the LC with SmA-N* phase transition was used and the resulting composite film could reversibly transverse a transparent and strong light-scattering state under temperature controlled conditions. More importantly, the film combined the advantages of large-area manufacturing and flexibility. Meanwhile, the dual-step polymerization was also carried out for an electrically controllable PD&PSLC composite film. The results showed that the demonstrated PD&PSLC composite film possessed better E-O properties and mechanical properties, besides good film formation.

## 4. Preparation of Nanoparticles Doped PDLC Composite Film

In conventional PDLCs, the LC, monomer and initiator are mixed. However, there are certain limitations to an all-organic system. Doping of nanoparticles (NPs) was attributed to the enhancement of optical, thermal and mechanical stability of the polymer matrix and interaction with the LC. NPs mainly influence the dielectric constant, refractive index or anchoring forces at the polymer/LC interface. Various types of NPs have been probed to prepare PDLC films with superior qualities. A few recent examples of the properties exhibited by NPs-doped PDLC are discussed below.

PDLC composite materials doped with ITO NPs were studied by various groups to optimize the films for applications in the field of energy saving due to their thermal insulation properties. In China, Wu et al. [71] of Nanjing University doped 1.5 wt% ITO powders of submicron size in the mixture (E7 + NOA65) and found that the operating voltages decreased due to the effects of ITO on the morphology of the PDLC as the anchoring force at the polymer/LC boundary was lowered. Recently Zhang et al. [72] of Xijing University, China, also used ITO NPs with acrylate-based monomers after surface treatment of NP with 3-methacryloxypropyltrimethoxysilan. However, they found that the driving voltage was increased and CR was decreased with the increment of doping content at 0–20 wt% of NPs loading, while the near infra-red (NIR) absorption property of films was enhanced in the wavelength range of 1300 to 2500 nm.

Silica NPs hold significant promise to improve the E-O properties of the PDLC [73]. In India, Jayoti et al. [74] of Dr. B. R. Ambedkar National Institute of Technology, Jalandhar, showed the effects of S-NPs on the morphology and E-O properties of PDLC by doping S-NPs into the PDLC based on NOA65 and BL036 LC. They reported a significant decrease in the driving voltage by doping 1.5 wt% S-NP and also transmittance of the films was enhanced. The NPs located at the LC-polymer interface modified the anchoring energy and thus effected the reorientation of LCs, which was attributed to the voltage drop. The dual size S-NPs was accredited to steric hindrances and resulted in slow phase separation during the photo-polymerization process. In Algeria, Beroguiaa et al. [75] of Université Aboubakr Belkaïd, reported that high concentration of S-NPs caused their aggregation at the LC polymer interface, which increased the anchoring energy and thus the reorientation voltage.

Other inorganic NPs such as ZnO, MgO, CuO, BTO, BaTiO_3_, Fe_3_O_4_, TiO_2_ [76,77,78,79,80,81,82] were doped into PDLCs and a decrease in driving voltage was observed. It was found that these particles tended to impact the dielectric constant of the medium or created local field effects which consequently lowered the driving voltage. The doping of ferroelectric NPs created spontaneous polarization which further improved the E-O properties [82,83]. In India, Mishra et.al [76] of Mumbai University, reported the fabrication of PDLC films by the SIPS method and embedded CuO, ZnO and Zn, NPs. A change in the structural and optical properties in the films was observed due to the gradient in the phase transition temperature confirmed by various characterization techniques such as Fabry Perot spectroscopic studies, optical polarized microscopy, differential scanning calorimetry, and Abbe refractometry using DSR Lamda. A change in the refractive index was noted because of the alteration of the orientational ordering properties of LCs and polymer networks due to the temperature increase.

In recent years, studies showed that metallic NPs-doped PDLC films exhibited better E-O, dielectric and optical properties. Particularly, Ag and Au-doped films possessed low driving voltages and high CR. This was attributed to surface plasmon excitations at metal-LC interfaces which enhanced the local electric fields [84,85]. A random lasing from dye-doped PDLCs containing Ag NPs was observed due to the cumulative effect of light scattering from nano-sized LC droplets and the local field enhancement around the silver NPs. In Taiwan, Chan et al. [86] of the National Central University, prepared core-shell particles by coating a thin layer of Ag on polystyrene microspheres and reported the effects of doping such particles on the operating voltage of PDLCs. The composite films contained LC (E7) and an acrylate-based polymer. They found that the induced electric field between the particles was enhanced upon the application of the external electric field and resulted in reduction of operating voltage from 77 V to 40 V.

Although the NPs have the potential to modulate the morphology of the films due to their intrinsic properties, it is difficult to predict how the NPs-doped PDLCs will perform relative to undoped PDLCs. In addition, the interaction of nanoparticles with the various recipes of the PLDCs is not easy to envisage and the performance comparison of NPs in a graphical or tabulated form to obtain the general sketch is of no use because the PDLC composition varies.

## 5. Preparation of Dye-Doped PDLC Composite Film

In dye-doped PDLCs (D-PDLCs), dye molecules were found to increase light absorption and decrease the power necessary for the optic effect [87]. D-PDLCs have been studied extensively by several groups, and it has been found that a strong microscopic mutual interaction among the dye and LC molecules influenced the LC refractive index, dielectric constant, orientational order and phase transition temperature [88,89,90,91,92]. E-O properties of the films were significantly improved by doping dyes due to their contribution to modifying the morphology of the PDLCs [93,94,95,96].

**Figure 5:**
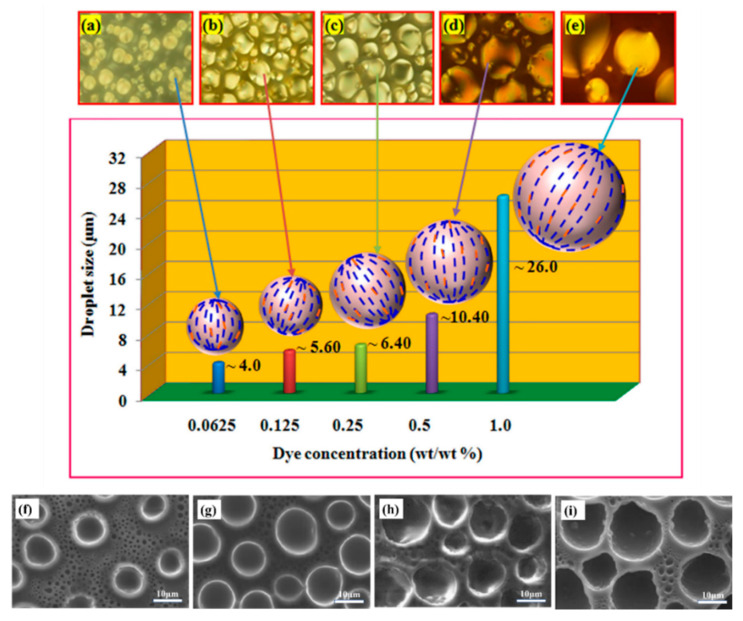
Graphical representations of LC droplets size of images observed by POM with 50× magnification taken during the experiment (**a**) bipolar and radial configuration of LC droplets and (**b**–**e**) stable bipolar configuration with respect to their diverse size of LC droplets with dye concentration. Reproduced with permission from ref. [97]. Copyright 2017, ELSEVIER Publications. SEM images of (**f**) pure PDLC, (**g**) PDLC + 1% MR, (**h**) PDLC + 3% MR and (**i**) PDLC + 5% MR composites. Reproduced with permission from ref. [87]. Copyright 2019, ELSEVIER Publications.

In India, Deshmukh et al. [94] of the Institute of Chemical Technology, Mumbai, reported the growth in the size of LC droplets at a higher concentration of orange dyes. They noticed that during photopolymerization, dye molecules absorbed some of the UV light available for polymerization and slowed down the phase separation process. Sharma et al. [97,98] of Chitkara University, Jhansla, Rajpura, India, also reported the incorporation of orange azo dichroic dye. Their finding revealed that higher dye concentration varied the transition temperature which transpired trans-cis photoisomerization of dye molecules as well as light absorption by the dye molecules to a slight extent. The azo dye molecule exhibited a rod-like shape in the trans form, but was bent and banana-like in the cis form, which weakened the intermolecular ordering interactions and order parameters of the LC. The polymerization rate was reduced since the vicinities were occupied by dye molecules, which resulted in increased LC droplet size as shown in Figure 5. The studies showed that the LC, polymer and dye were chosen such that the solubility of dye in the polymer was low but high in the LC.

## 6. Preparation of Carbon Nanofillers-doped PDLC Composite Film

Carbon nanofillers such as nanographite, fullerenes and carbon nanotubes (CNTs) have been paid more attention due to the advantages of their high electric, thermal and mechanical properties. Specifically, the alignment of CNTs is crucial for anisotropic mechanical and electric properties: their properties are extraordinarily large along the tube axis and small along a perpendicular direction. Single wall nanotubes (SWNTs) and multiwall nanotubes (MWNTs) are two representative types of CNTs successfully applied as dopants to PDLCs to improve their E-O performance.

Jain et al. [99] of the Institute of Chemical Technology, Mumbai, India, prepared two types of MWCNT-doped PDLCs (CPDLC), C00N and C36N using mixtures of two different LCs (LC BL036 and LC HPC850100-100), and NOA65. The LC droplet size was not influenced by the doping concentration because no UV light was absorbed by MWCNT. At the optimal doping concentration (0.005%) CR and threshold voltage of C36N, CPDLC composite film was 990 and 1 V, respectively, and that of C00N CPDLC composite film was 137 and 0.1 V, respectively. However, the composite film shrank because of the formation of conductive channels between two electrodes of the composite film with the increment of doping concentration. For the relaxation behavior, the two composite films both showed Debye-type behavior having a zero value of the distribution parameter. Wu et al. [100] from the University of Science and Technology, Beijing, China, focused on the improvement of E-O properties of PDLC composed of acrylate monomers, SLC1717, and MWCNT modified by a cyanobiphenyl functional group. The results indicated a reduction of driving voltage at an optimal MWCNT doping content of 0.01–0.03 mg, which was attributed to the enhancement of the electric field by reducing the resistivity of the medium and the increase of the capacitance of the cells induced by the MWCNT. Moreover, the dispersion of MWCNT was further improved through modification, and the corresponding anchoring effect on the LC molecules was weakened, which improved the transmittance.

## 7. Applications

The efforts dedicated to fabrication methods and various outstanding properties make PDLCs suitable for promising applications in energy saving smart windows, photovoltaics, optical elements, light shutters, switching gratings, sensors, microlenses, lasers, smart food packaging, electrically switchable high-fold-helix spiral phase plates and biocompatible materials, as well as biomedical devices. In this section, we presented the information on the aforementioned applications in detail.

### 7.1. Traditional Applications

Since the beginning, the electrically switchable peculiarity of PDLCs from a light scattering state to a transparent state has led to their excellent performance and extended application prospects in energy-efficient smart windows. Although the fabrication of PDLC-based windows has not changed in recent years, new formulations suitable for this purpose have been investigated thoroughly. Particularly, the fundamental bottleneck was the optical modulation confined within a limited waveband, either visible (400–800 nm) or near-infrared (NIR, 800–2500 nm) region. The shorter wavelength invisible NIR (800 to 1500 nm) carries about 50% of entire solar energy and, therefore, the shielding performance of a smart window in this range is crucial. To develop a highly efficient energy-saving smart window Liang et al. [101] recently demonstrated the broadband optical modulation, the regulation of visible light transmittance [102] and the shielding performance of NIR light from 800 nm to 2500 nm in PDLC films. They combined a poly(vinylpyrrolidone) (PVP) tuning layer between tungsten bronze (Cs_x_WO_3_) nanorods (NRs) and a polymeric syrup containing liquid crystals with a smectic A (SmA) to chiral nematic (N*) phase transition, followed by forming an elaborately designed polymer structure within the film [103]. In essence, the field of the PDLC for smart windows is still evolving [104,105,106] and there is still huge room for improvement.

Transparent displays based on PDLCs have high visibility and require a black color as well as complete blocking of the background. In Korea, Yoon et al. [107,108] of the National University, Busan, prepared devices which consist of a dye-doped cholesteric liquid crystal (ChLC) layer and a polymer dispersed liquid crystal (PDLC) film (E7 and NOA 65), for simultaneous control of haze and transmittance. In the opaque state, this can not only provide a black color by using the dye-doped ChLCs but also hides the objects behind the display panel by using the PDLC film. The proposed light shutter shows a high haze value of 90.7% with a low specular transmittance of 1.20%.

### 7.2. HPDLC

Holographic PDLCs (HPDLCs) are another class of polymer-LC composite wherein prepolymer/ monomer/oligomer concentrations are quite high compared to PDLC. HPDLC structures (periodic dark and bright fringes) are created in an LC cell filled with a mixture of LC, monomer and photoinitiator (PhI) exposed under the interference pattern generated by two, or multiple, coherent laser beams with proper wavelength and power. The interference pattern formed gives rise to nonuniform photopolymerization and phase separation. High polymerization rate in the bright region (diffusion of the monomer from dark to bright region) and low polymerization rate in dark region (diffusion of LC from bright to dark region) creates a Bragg grating with alternate polymer-rich and LC-rich regions. A periodic refractive index profile is formed in the cell, and thus light can be diffracted. Morphology and diffraction properties of the grating depend upon writing set-up, materials, diffusion rate, curing conditions and the phase separation process Typical morphology, the working principle and electro-optical properties of HPDLCs are thoroughly elaborated in chapter 5 of the recently published book [109]. Promising applications of HPDLCs include diffraction gratings (DGs), polymer slices (POLICRYPS), waveguides, autostereoscopic image splitter, three-dimensional (3D) data storage and photonic crystals [110,111,112,113,114,115,116,117].

Despite several attempts to overcome the need for a strong electric field when the diffraction effects are not required, E-O efficiency of the HPDLCs was compromised. In the United States, TJ. Buning et al. [118] of the Beam Engineering for Advanced Measurements Company, Florida and Air Force Research Laboratory, Wright-Patterson Air Force Base, Dayton, Ohio, demonstrated highly-efficient and fast DGs which were invisible (transparent) in the off state and exhibited strong diffractive properties upon the application of a moderate E-field by employing photocurable LC monomer and nematic LCs using holographic photopolymerization techniques. Similarly, Manda et al. [119] of Chonbuk National University, Korea, obtained DGs which were optically transparent in the visible wavelength regime by tuning the LC droplet size below the visible wavelength. Efficient HPDLCs having low driving voltages and high diffraction efficiency were realized by using a photoinhibitor, acrylamide and doping ZnS nanoparticles [120,121]. Also, a concurrent photoinitiation and inhibition upon green light illumination was disclosed, which significantly improved the diffraction efficiency of HPDLC gratings and afforded their utilization in storage of colored 3D images [122].

### 7.3. Novel Films

**Figure 6:**
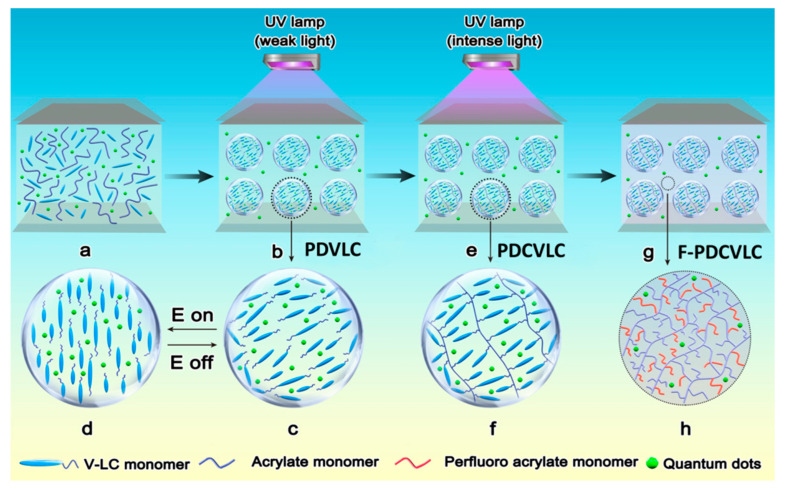
Schematic illustration of the preparation of crosslinked V-LCs/polymer composite films. (**a**) The homogeneous polymeric syrup containing quantum dots sandwiched between two conductive ITO glass substrates coating with PVA. (**b**) The PDVLC film was formed by the first step radical polymerization of weak UV light to initiate microphase between V-LCs and polymer matrix. (**c**) Enlarged V-LCs domains with randomly oriented liquid crystal mixture. (**d**) V-LCs were homeotropically aligned when the electrical field was applied. (**e**) The second step cationic polymerization of intense UV light was carried out to prepare the PDCVLC by the polymerization of V-LC monomers. (**f**) Enlarged V-LCs domains with the polymerization of V-LC monomers. (**g**) The F-PDCVLC film was developed by introducing the perfluoro acrylate monomers into the original syrup and repeating the above dual-step polymerization. (**h**) Enlarged polymer matrix with the perfluoro acrylate monomers. Reproduced with permission from ref. [8]. Copyright 2019, ELSEVIER Publications.

Although the displays based on PDLC films are simple and bright, there is huge room for improvement. In this regard, optical diffusers play a critical role in preventing light sources from being seen directly by viewers in the lighting system. Additionally, optical diffusers are a key optical component in light-emitting diodes (LEDs), which can spread the point light source uniformly without high intensity spots. In our group Zhou et al. [7,123,124,125,126] designed optical diffusers with very high efficiency. Recently, an optical diffuser was prepared via a thiol ene click reaction comprising of 1:1 of ene and thiol and exhibited high transmission (98.49%) and high haze (91.77%). Similarly, display devices with narrow viewing angle (NVA) and wide viewing angle (WVA) characteristics for privacy protection and information sharing were demonstrated [127]. Han et al. [128] from the University of Science and Technology, Beijing, China, fabricated a controllable antipeeping device with a laminated structure of microlouver and PDLC films which reversibly switched between a wider viewing angle range (VAR) for the WVA mode and a narrower viewing angle range (VAR) for the NVA mode at 0 V and 8 V, respectively. In our recent studies novel QDs films based on a liquid crystals/polymer composite were developed [8,129]. The fluorescence enhancement of QDs-doped TiO_2_/polymer, LCs/polymer and TiO_2_/LCs/polymer composite systems were comparatively studied. The photoluminescence enhancement of QDs was induced by the synergistic effect of LCs/polymer composite and TiO_2_ nanoparticles (150 nm particle size) having 0.1 wt% loading content. The enhancement factor of QDs-doped TiO_2_/LCs/polymer composite film was approximately 6-fold relative to the QDs-doped polymer. The polymer dispersed vinyl-ether liquid crystals (PDVLC), polymer dispersed crosslinked vinyl-ether liquid crystals (PDCVLC) and fluorine-containing polymer-dispersed crosslinked vinyl-ether liquid crystals (F-PDCVLC) QDs films were fabricated by dual-step polymerization as shown in Figure 6. The reported PDVLC film had fluorescence enhancement and tuning, whereas the PDCVLC film not only maintained fluorescence enhancement of the PDVLC film but also improved its water-resistant performance. Similarly, hydrophobic PDLC films for ecofriendly optoelectronic applications were demonstrated by the encapsulation of cholesteryl acetate LC by a chitosan polymer matrix [130].

### 7.4. Device Components

PDLCs have been used as device components for OLED, Micro-LEDs, solar power collector, OFET, and solar cells. In United States, D.K.Yang et al. [9] of Kent State University, Ohio, demonstrated PDLC films in which LC droplets were unidirectionally aligned under an applied electric field along the film normal direction. This PDLC film was used to enhance the light outcoupling efficiency of OLEDs, because it could selectively scatter light emitted from the OLED into directions with large incident angles but not light with small incident angles, and thus reduced light loss due to total internal reflection. Similarly, Wu et al. [131] of the University of Central Florida, USA, demonstrated volumetric light-shaping PDLC films for mini LED-backlit liquid crystal displays (LCDs). PDLC films with a thickness 50-μm were prepared without surface alignment using a mixture containing LC (ZLI 1844 = 49.92%) and prepolymer (NOA 60 = 47.11%). With proper material engineering, passive PDLC films with angle-selective scattering properties were shown which respond to angles rather than spatial locations.

**Figure 7:**
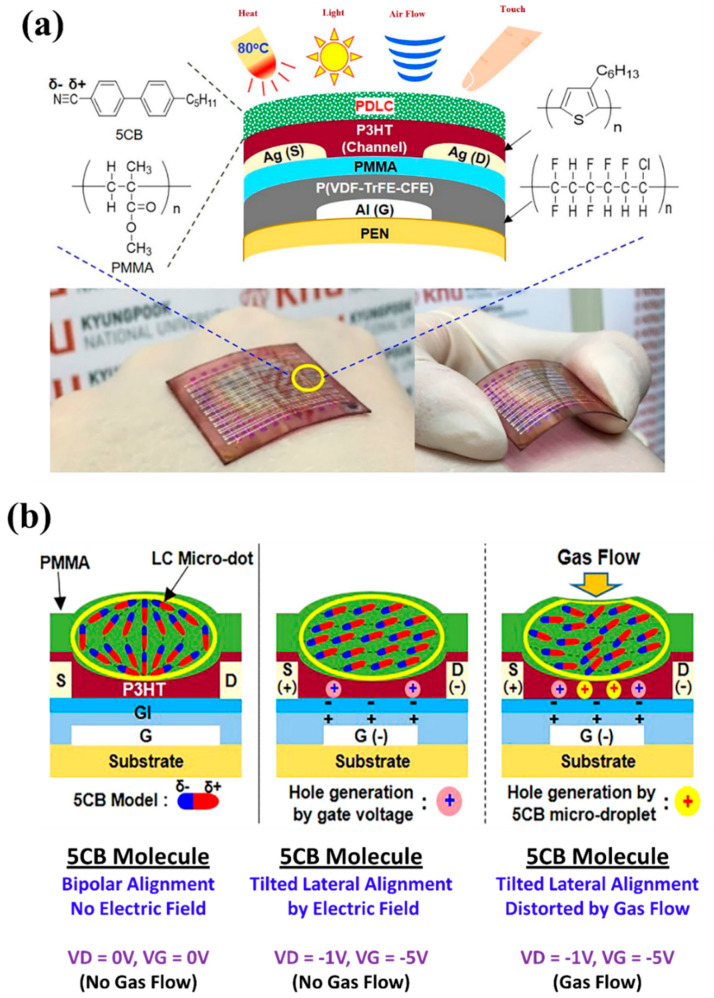
Structure and basic device performances of flexible PDLC-i-OFETs. (**a**) Illustration for the device structure and materials used in this work (photographs demonstrate attachment of flexible PDLC-i-OFET array sensors on human hands), (**b**) Sensing performance by weak gas flow stimulations and mechanism change of 5CB alignment in the channel region of PDLC-i-OFET devices according to applied voltages and nitrogen gas stimulations: illustrations for possible orientation of 5CB molecules in the LC micro-dots in the channel layers. Reproduced with permission from ref. [133] Copyright 2017, Springer Nature Publications.

Monolithic integration of CNT thin-film transistor driver circuits with PDLC pixels has been studied [132]. The fabricated PDLC pixels possessed good contrast and high-performance. In Korea, Song et al. [133] of the Kyungpook National University, Daegu, demonstrated PDLC integrated-organic field-effect transistors (PDLC-i-OFETs). The PLDC sensing layers were prepared by embedding 4,4′-pentyl-cyanobiphenyl (5CB) microdots in the poly (methyl methacrylate) (PMMA) matrix, which were integrated on the flexible OFETs with 200 μm-thick poly (ethylene naphthalate) (PEN) substrates as shown in Figure 7. The PDLC layers were found to contain well-aligned LC micro-dots embedded in the PMMA layers. The fabricated PDLC-i-OFET devices were able to sense four different stimulations such as weak air (gas) flow, strong physical force (touch), light and heat, as shown in Figure 7b. The concept of combining PDLC and OFET could contribute to the further development of multifunctional organic sensory devices for various applications including humanoid robots, artificial skin and wearable sensors.

**Figure 8:**
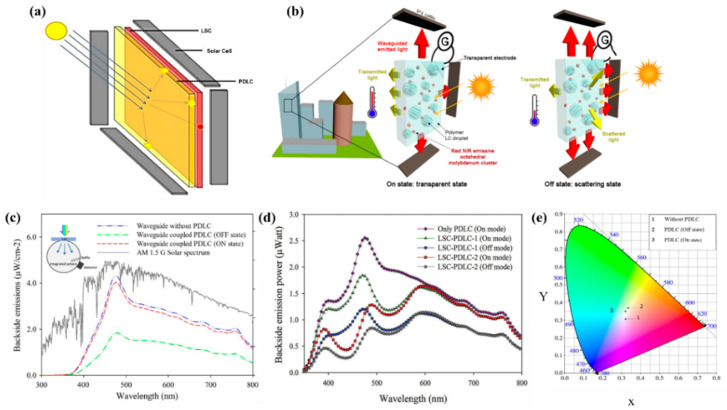
(**a**) Schematic representation and working principle of PDLC-coupled LSC [138] (**b**) Schematic exploded representations of an active LSC window containing red-NIR phosphorescent octahedral molybdenum nanoclusters composed of a PDLC matrix containing the phosphorescent inorganic emitter surrounded by PV cells on its edges [138]. Figure 8a,b reproduced with permission from Ref. [138] Copyright 2017, ACS Publications (**c**) PDLC backside emissions of an N-CQDs-based waveguide coupled with no PDLC and with PDLC. The driving voltage in the ON state was kept constant at 50 V (**d**) Backside emission power of PDLC-LSC devices in the OFF and ON mode. The driving voltage in the ON mode was kept constant at 50 V in both devices. (**e**) Color possibilities of transmitted light through the ON and OFF states of a waveguide-coupled PDLC device plotted on a CIE 1931 XY chromaticity diagram. Figure 8c–e reproduced with permission from Ref. [134,136]. Copyright 2019, ELSEVIER Publications.

Conventional PDLCs-based windows require external electricity to operate, and these devices cannot be combined with traditional solar cells for energy savings. To achieve nearly zero energy-consuming windows, luminescent solar concentrators (LSCs) were integrated with PDLC devices [134,135,136,137]. The schematic illustration of LSC-coupled PDLC is shown in Figure 8a,b. An important aspect of the LSC device is its capability to harvesting direct, diffused and ground-reflected light. Therefore, a measurable amount of energy can be generated even in nonideal illumination conditions. In Korea, Mateen et al. of Dongguk University, coupled an LSC based on luminophores (MACROLEX Fluorescent Yellow 10GN and MACROLEX Fluorescent Red G (Bayer, Germany)) and PDLC (E7 and NOA 65). The PDLC was placed on the backside of the LSC to reduce the backside light losses of LSC because PDLC in off mode acted as a backside scattering reflector as shown in Figure 8b–d. PDLC film not only reflected the untrapped light into the LSC part but also redirected the light that was not absorbed (i.e., outside the absorption range of the luminophores) towards the edges, and thus to the attached solar cells. The electricity produced by the LSC part was successfully employed for the switching operation of the PDLC device.

### 7.5. Others

The application of PDLC composites in biomedical devices has shown significant promise [139,140]. In Romania, Marin et al. [141,142,143] from the Institute of Macromolecular Chemistry, prepared ecofriendly PDLCs for sensing applications by embedding biocompatible LCs in the polymer matrix of polyvinyl alcohol-boric acid (PVAB). The composites films exhibited well-defined droplets of submicrometric size (250–650 nm). It was found that the composite films possessed moderate wettability and showed selective responsiveness for L or D sugars, amino acids and DNA.

PDLCs were also shown to be sensitive to ultrasound through the acousto-optic effect. In the United Kingdom, Edwards et al. [144] of the University of Warwick, Coventry, prepared a PDLC sensor (E7: NOA68= 75:25) and studied the acousto-optic response of PDLCs coupled with an ultrasound transducer over a broad frequency range (0.3–10 MHz). The displacements required to produce acousto-optic clearing of PDLC films were between around 2 and 50 nm. These frequency dependent displacements could be used for ultrasound visualization in nondestructive testing. Recently, Firoozi et al. [145] reported a harmonic response in PDLCs to an applied square-wave voltage at low frequencies (4.5–5.0 Hz) enabling their use in interferometers. Another important application of the PDLCs is a polarization-dependent (Micro lenticular lens array) MLA device [146]. This device was used to realize a 3D image when the polarized 2D image arrays encountered the microlens [147].

## 8. Conclusions

As detailed in this review, significant achievements have been made in the preparation, properties and applications of polymer dispersed liquid crystal composite films between 2014 and 2020. The electro-optical properties of the PDLC films were optimized by the structural variation of monomers and liquid crystals, two-step polymerization as well as doping nanoparticles, dyes and CNTs. Specifically, the two-step polymerization paved a new way to improve the electro-optical and mechanical performance of PDLC composite films. Meanwhile, significant progress in applications was also achieved such as diffuser films, antipeeping films, QDs films, device components of OLED, OFET, sensors, photovoltaics, energy harvesting, actuators, biomedical applications, etc. Featuring the advantages of simple preparation, cost-efficiency, large-area manufacturing and flexibility, PDLC composite films promise to promote and expand the development of next-generation smart windows, displays, wearable devices and sensors.

## Figures and Tables

**Table 1:** Some (meth)acrylate monomers used for the fabrication of various PDLC composites.

No.	Monomers	Chemical Structure	Reference
1	Lauryl acrylate	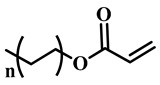	[48]
2	Lauryl methacrylate	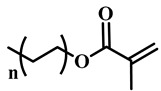	[50]
3	Hydroxypropyl methacrylate	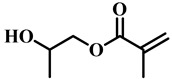	[49,50]
4	3, 5, 5-trimethelhexyl acrylate	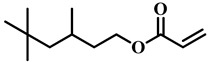	[51]
5	Glycidyl methacrylate	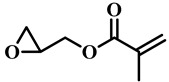	[49]
6	Tetrahydrofurfuryl acrylate	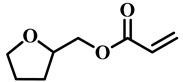	[48]
7	Tetrahydrofurfuryl methacrylate	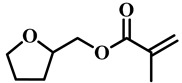	[48,49]
8	Benzyl methacrylate	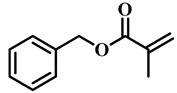	[48,49]
9	Cyclohexyl methacrylate	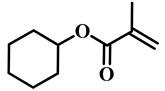	[48,49]
